# A founder mutation in *CA5A* causing intrafamilial and interfamilial phenotypic variability in a cohort of 18 patients with carbonic anhydrase VA deficiency

**DOI:** 10.1002/jmd2.12426

**Published:** 2024-05-08

**Authors:** Khalid Al‐Thihli, Nadia Al Hashmi, Aaisha Al Balushi, Asila Al‐Habsi, Eiman Al‐Ajmi, Fatma Al‐Jasmi, Fathiya Al‐Murshedi

**Affiliations:** ^1^ Genetic & Developmental Medicine Clinic, Department of Genetics Sultan Qaboos University Hospital Muscat Oman; ^2^ Genetics Department, College of Medicine and Health Sciences Sultan Qaboos University Muscat Oman; ^3^ Department of Pediatrics the Royal Hospital Muscat Oman; ^4^ Department of Nursing Sultan Qaboos University Hospital Muscat Oman; ^5^ Department of Radiology and Molecular Imaging Sultan Qaboos University Hospital Muscat Oman; ^6^ Department of Genetics and Genomics, College of Medicine and Health Sciences United Arab Emirates University Al Ain United Arab Emirates; ^7^ Department of Pediatrics Tawam Hospital Al Ain United Arab Emirates

**Keywords:** *CA5A*, carbonic anhydrase‐VA, CA‐VA, founder mutation, hyperammonemia, hyperCkemia, hypoglycemia, lactic acidemia

## Abstract

Carbonic anhydrase VA (CA‐VA) deficiency is a rare cause of hyperammonemia caused by biallelic mutations in *CA5A.* Most patients present with hyperammonemic encephalopathy in early infancy to early childhood, and patients usually have no further recurrence of hyperammonemia with a favorable outcome. This retrospective cohort study reports 18 patients with CA‐VA deficiency caused by homozygosity for a founder mutation, c.59G>A p.(Trp20*) in *CA5A*. The reported patients show significant intrafamilial and interfamilial variability, and display atypical clinical features. Two adult patients were asymptomatic, 7/18 patients had recurrent hyperammonemia, 7/18 patients developed variable degree of developmental delay, 9/11 patients had hyperCKemia, and 7/18 patients had failure to thrive. Microcephaly was seen in three patients and one patient developed a metabolic stroke. The same variant had been reported already in a single South Asian patient presenting with neonatal hyperammonemic encephalopathy and subsequent development of seizures and developmental delay. This report highlights the limitations of current understanding of the pathomechanisms involved in this disorder, and calls for further evaluation of the possible role of genetic modifiers in this condition.

**Synopsis**

CA‐VA deficiency results in great phenotypic heterogeneity and clinical outcomes that calls for careful follow up of patients and further evaluation for possible genetic modifiers.

## 1 INTRODUCTION

Carbonic anhydrase VA (CA‐VA) deficiency (OMIM# 615751) is a rare autosomal recessive disorder caused by biallelic mutations in *CA5A*.^1^, ^2^, ^3^, ^4^ While the mitochondria are impermeable to bicarbonate, CA‐VA plays a pivotal role to provide bicarbonate to four mitochondrial enzymes involved in intermediary metabolism, namely carbamoylphosphate synthetase 1, pyruvate carboxylase, propionyl‐CoA carboxylase, and 3‐methylcrotonyl‐CoA carboxylase.^5^, ^6^ Affected patients with CA‐VA deficiency usually present with lethargy, hyperammonemic encephalopathy with variable hypoglycemia, metabolic acidosis, ketosis, and lactic acidosis of early infantile or childhood onset.^1^, ^2^, ^3^, ^4^, ^7^, ^8^ Preliminary reports suggest that hyperammonemia caused by CA‐VA deficiency responds well to carglumic acid. Most patients reported so far had no further episodes of decompensations or metabolic crisis beyond the first episode that brought them to medical attention.^1^ The prognosis of developmental outcome has mostly been favorable,^3^, ^8^ however, some patients have been reported with developmental delay or delayed motor skills, while intractable hyperammonemia with fatality has also been rarely reported.^9^

This retrospective cohort study reports clinical, biochemical, and molecular features of 18 patients with CA‐VA deficiency caused by a founder mutation, c.59G>A p.(Trp20*) in *CA5A*, belonging to 10 different families pertaining to five different tribes from the same mountain region of the Arabic peninsula. The same mutation had been reported previously in a South Asian patient with a phenotype that included neonatal encephalopathy with hypoglycemia, elevated ammonia, and lactate, and subsequent development of infantile spasms at the age of 8 months.^10^

This study describes the largest single cohort of patients with CA‐VA deficiency reported so far; it reports patients with recurrent metabolic crisis, poor developmental outcomes as well as patients who remain asymptomatic into adulthood. The study also emphasizes the already recognized^1^, ^3^, ^4^, ^9^, ^10^ significant intrafamilial and interfamilial phenotypic and prognostic variability associated with the same founder mutation.

## 2 METHODS

This is a retrospective review of all patients diagnosed with CA‐VA deficiency at Sultan Qaboos University Hospital (SQUH) and the Royal Hospital (RH) at the Sultanate of Oman, and Tawam Hospital (TH) at United Arab Emirates since the first patient diagnosed and until end of June 2023. All patients confirmed to have CA‐VA deficiency through identification of biallelic disease causing variants in *CA5A* were included in this study without age restriction. Causative variants were identified through whole exome sequencing or target mutation testing for the most recurrent variant (founder mutation) identified in this study. Demographic and clinical characteristics were reviewed through electronic patients records, and the data collected was anonymized and coded. Descriptive statistics were used for data representation. The study was approved by the Medical Research Ethics Committee at Sultan Qaboos University Hospital (MREC# 3231).

## 3 RESULTS

A total of 18 patients were diagnosed with CA‐VA deficiency at the three sites involved in this study, 15 at SQUH, 2 at RH and 1 at TH. All of them were confirmed to be homozygous for c.59G>A p.(Trp20*) variant in *CA5A*. The variant was identified either through whole exome sequencing (WES) or through testing for the known founder variant as outlined in Table 1. Patients belong to 10 different families representing five different tribes; 11 were females and 7 were males. At the time of reporting patients' ages ranged between 10 months and 30 years, with a median of 5 years; 16/18 (88.9%) patients were symptomatic, 13/16 (81.3%) patients presented in the first week of life, with the age of presentation ranging between 30 h of life and 5 years of age, and a median of 3 days; 2/18 (11.1%) patients were asymptomatic adults with normal intellect who were identified during familial segregation studies carried out during evaluation of the variant for pathogenicity at the time it was classified as a novel variant of uncertain significance.

**TABLE 1:** Demographic and clinical characteristics and outcomes of the 18 patients with carbonic anhydrase VA deficiency due to homozygous c.59G>A; p.(Trp20*) variants in *CA5A* gene.

Tribe	Family/patient	Gender	How the mutation was detected	Age at presentation	Highest ammonia^a^ (μmol/L) (15–60)	Duration of hyperammonemia	Highest lactate^a^ (mmol/L) (0.5–2.2)	Ketonuria^a^	Hypoglycemia^a^ (mmol/L)	CK	Age at last clinical assessment (years)	Neurodevelopmental outcome	Failure to thrive	Microcephaly
T1	F1/P1	M	WES	1 day	774	2 days	15.1	No	No	813	5	GDD	Yes (−2.4 SD)	Yes (−7 SD)
	F2/P2	F	WES	4 days	964	2 days	5.3	No	2.4	730	5	Severe GDD	Yes (−2.7 SD)	Yes (−3 SD)
	F2/P3^b^	F	Targeted gene sequencing	3 days	1300	3 days	10.3	No	0	658	3	GDD	Yes (−3.5 SD)	No
	F2/P4^b^	M	Targeted gene sequencing	2 days	465	1.5 days	10.8	No	NA	NA	1	Normal	Yes	No
	F3/P5	M	Targeted gene sequencing	3 days	703	2 days	4.4	No	NA	345	4	Mild ID	No	No
	F4/P6	F	Targeted gene sequencing	3 days	400	4 days	2.6	No	NA	81	3	Normal	No	No
	F5/P7	F	Targeted gene sequencing	2 days	350	NA	NA	NA	NA	NA	7	Normal	Yes (−3 SD)	No
	F5/P8	F	Targeted gene sequencing	3 days	300	NA	NA	NA	NA	NA	3	Normal	No	No
	F6/P9	F	Targeted gene sequencing	5 years	446	2.5 days	8.3	No	1.6	NA	5	GDD	Yes	Yes (−3 SD)
T2	F7/P10	M	WES	2 days	363	1.5 days	13	No	No	600	8	Normal	No	No
	F7/P11	F	Targeted gene sequencing	3 days	689	NA	12.5	Yes	No	525	4	Normal	No	No
	F7/P12	F	Targeted gene sequencing	3 days	NA	NA	NA	NA	NA	449	14	Normal	No	No
	F7/P13	M	Targeted gene sequencing	3 days	NA	1.5 days	NA	NA	NA	364	17	Normal	Yes (−2.9 SD)	No
T3	F8/P14	F	Targeted gene sequencing	3 days	450	1 day	6.4	NA	NA	NA	13	Normal	No	No
	F8/P15	F	Targeted gene sequencing	NA	NA	1 day	NA	NA	NA	NA	27	Normal	No	No
	F8/P16	M	Targeted gene sequencing	NA	NA	3 days	NA	NA	NA	NA	30	Normal	No	No
T4	F9/P17	M	Targeted gene sequencing	4 years	243	1 day	3.8	Yes	No	60	4	GDD	Yes	No
T5	F10/P18	F	WES	18 months	671	13 h	9.8	Yes	0	616	14	GDD	No	No

*Note*: Values in between brackets represent reference intervals.

Abbreviations: CK, creatine kinase; GDD, global developmental delay; ID, intellectual disability; NA, not available; SD, standard deviation; WES, whole exome sequencing.

^a^ These denote values or observations encountered during the first presentation.

^b^ Concurrent diagnosis of CA‐VA with medium chain acyl‐coenzyme A dehydrogenase (MCAD) deficiency.

Of the 16/18 (88.9%) patients who were symptomatic, 15/16 (93.8%) had acute encephalopathy except for the patient with preemptive diagnosis driven by family history. Ammonia measurements during first presentation were available in 16/18 (88.9%) patients whereas the two asymptomatic patients had normal ammonia. Of the remaining symptomatic 14 patients, ammonia was elevated with the level ranging between 120 and 1300 μmol/L, with a median of 458 μmol/L. Hyperammonemia lasted between 1 and 4 days in the 14 patients. Medications used included the ammonia scavengers sodium benzoate and sodium phenylbutyrate, and the pharmacological activator of CPS1, carglumic acid. When carglumic acid was used alone in three patients to treat hyperammonemia based on knowledge of founder effect or family history, ammonia normalized in less than 24 h. None of the patients required continuous renal replacement therapy, and there was no death because of hyperammonemia. However, recurrent hyperammonemia was seen in 7/18 patients (38.9%), with acute gastroenteritis precipitating recurrence in three of them. Hypoglycemia was documented in 4/16 patients (25%), and ketosis was reported in 3/12 (25%) patients. Lactate measurements were available in 14/16 (87.5%) symptomatic patients, and 2/14 (14.3%) had normal lactate, while lactate was elevated in 12/14 (85.7%) patients with measured lactate ranging between 2.6 and 15.1 mmol/L. Creatine kinase (CK) was measured in 11 patients, and 9/11 (81.8%) patients had mildly elevated CK ranging between 345 and 813 U/L, with a median of 592. Plasma amino acids were quantified in eight patients, and 6/8 (75%) showed clinically unremarkable amino acids profile except for findings reflective of reduced protein intake, while 1/8 (12.5%) showed significantly elevated glutamine with hyperalaninemia and hyperprolinemia in addition to mildly elevated methionine and one patient showed isolated mildly elevated ornithine. Urine organic acids were evaluated in three patients, one showing essentially unremarkable profile while the other two showed lactic aciduria with mildly elevated 3‐hydroxoisovaleric acid and markers of ketosis. Severe global developmental delay was seen in 2/18 (11.1%) patients, while mild to moderate global developmental delay was evident in 5/18 (27.8%) patients, and the developmental outcome was normal in 11/18 (61.1%) patients.

**FIGURE 1:**
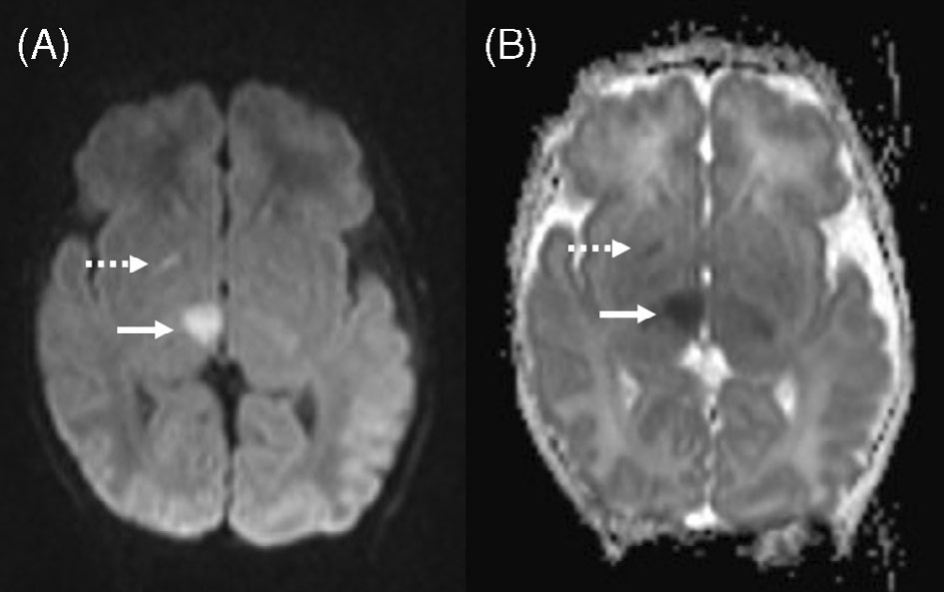
MRI of patient 1 at the age of 3 days: (A) diffusion weighted image and (B) ADC map show focal diffusion restriction in right basal ganglia (dashed arrow) and right thalamus (solid arrow).

**FIGURE 2:**
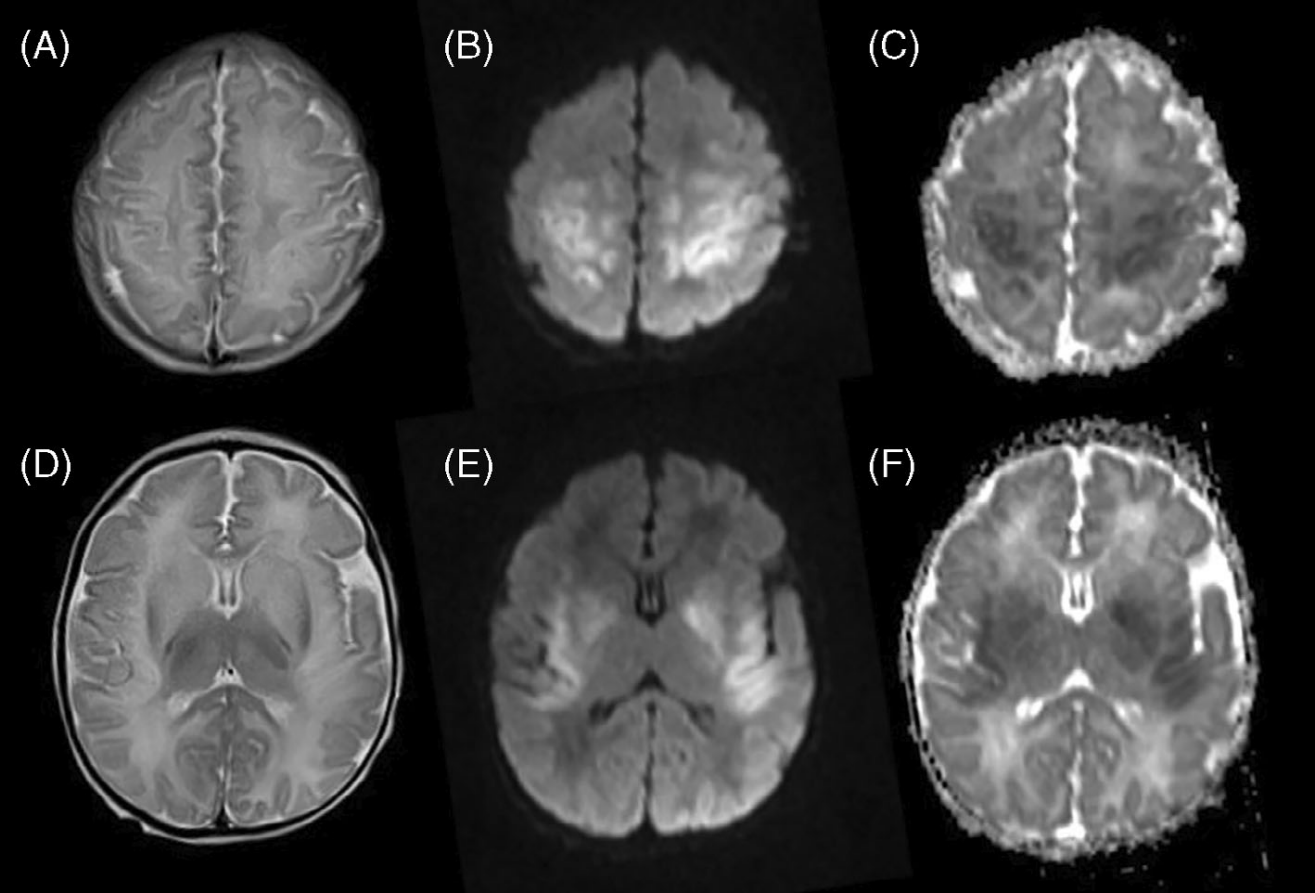
MRI of patient 2 at the age of 9 days: (A and D) axial T2‐weighted images, (B and E) diffusion weighted images, and (C and F) ADC maps show bilateral symmetric faint abnormal T2 signal intensity and diffusion restriction in the perirolandic cortex (images A–C) and in the insula and basal ganglia (images D–F).

Chronic hyperammonemia was not demonstrated and all patients had normal ammonia while asymptomatic during their clinic visits except for one who had mild hyperammonemia (94 μmol/L) and mildly elevated lactate (4.3 mmol/L) while asymptomatic during a routine follow up visit. During the first episode of hyperammonemic encephalopathy at the age of 3 days, MRI brain in patient 1 showed mild brain edema with focal areas of diffusion restriction in the right lentiform nucleus and right thalamus in keeping with acute infarcts (Figure 1). MRI brain in patient 2 at the age of 9 days showed abnormal T2 signal intensity and diffusion restriction in the perirolandic cortex, insula, and basal ganglia in keeping with hyperammonemic encephalopathy (Figure 2). MRI brain in patient 3 showed changes consistent with hyperammonemic encephalopathy as well while CT brain was normal in three other patients. Failure to thrive was encountered in 7/18 (38.9%) patients, and 3/18 (16.7%) patients had microcephaly, with the latter being most severe in patient 1 (−7 SD). This patient was diagnosed through WES, and exome reanalysis until January 2024 did not reveal another explanation for his severe microcephaly. Two siblings have concurrent diagnosis of medium chain acyl coenzyme A dehydrogenase deficiency (MCAD), diagnosed in the context of neonatal hypoglycemia and typical acylcarnitine abnormalities, one confirmed via WES and the other through targeted gene sequencing. Table 1 summarizes the demographic characteristics, biochemical features, and clinical outcomes reported in this cohort.

## 4 DISCUSSION

This study describes the clinical characteristics and outcomes in the largest cohort of patients with CA‐VA deficiency reported to date in a single study. The patients are from five different tribes originating from the Oman Hajar mountains (the highest mountain range in the eastern part of the Arabian Peninsula, shared between northern Oman and eastern UAE), and all of them were proven to be homozygous for the same c.59G>A p.(Trp20*) variant in *CA5A*, arguing for a founder effect. This variant was previously reported in an infant from South Asian ethnic background who developed irritability, poor feeding, and hypotonia on the third day of life and found to have hypoglycemia, hyperammonemia of 379 μmol/L, and elevated lactate. He progressed then to treatment refractory seizures and required mechanical ventilation for 10 days. Antiepileptic medications were stopped gradually but the infant presented at the age of 8 months with infantile spasms that were resolved by adding topiramate. His outcome was complicated by microcephaly and developmental delay as he can only partially support his neck at the age of 14 months.^10^ A similar clinical presentation with neonatal treatment refractory seizures is noted in one patient only (patient 1, Table 1) of our cohort. However, the reminder of patients we report in this study show significant intrafamilial and interfamilial variability that is unexplained by the degree of hyperammonemia, age of presentation, or associated morbidities. The range of complications seen in this cohort relative to the previously reported favorable outcomes seen in patients with CA‐VA deficiency^2^, ^4^, ^7^, ^8^ calls for caution with prognostic implications of this diagnosis.

Although earlier published studies suggest that patients affected with CA‐VA deficiency usually do not develop further episodes of hyperammonemia beyond the first episode that brings them to medical attention,^1^ recurrence of hyperammonemia was reported in a patient with CA‐VA deficiency who experienced seven hyperammonemic episodes over a 3‐year period, up to age of 5 years 9 months.^11^ In agreement with this observation, 7/18 patients have developed recurrent hyperammonemia in the context of physiologic stress exemplified with febrile illness or acute gastroenteritis; 11/18 indeed experienced no further episodes of hyperammonemia in agreement with the previously published observations. Absence of further episodes of hyperammonemia in affected patients despite the severe hyperammonemia seen in some of them at presentation remains unexplained, and the possibility of underreporting of recurrent hyperammonemia in this disorder cannot be excluded. Similarly, why some of the patients we report here developed recurrent hyperammonemia despite having the same causative genetic variant shared with other patients in this cohort remains unexplained. Arguably, recurrence of hyperammonemia in patients with CA‐VA deficiency is multifactorial in nature, and the observation we make here potentially impacts counseling, sick day management, and monitoring of these patients. Vigilance and awareness of this complication might be a safe practice to adhere to when caring for these patients.

On the other hand, 2/18 patients in this cohort were asymptomatic adults at the time of evaluation without previously documented symptomatic hyperammonemic episodes despite sharing the same homozygous variant presenting with severe neonatal presentation in most other patients. Although this may fall within the spectrum of disease presentation as seen in the variability of age of onset displayed in some patients, it would be difficult to assume that a precipitant that may lead to a crisis in these adult patients has never been encountered throughout their lives. This observation has already been made even in the original report of the first patients that lead to discovery of this condition as one of the patients reported had no apparent phenotype.^4^ These observations synergize to argue that this condition is subjected to the influence of genetic modifiers. A likely possible genetic modified in this disease is carbonic anhydrase VB (CA‐VB), the only other carbonic anhydrase with mitochondrial localization.^5^, ^12^, ^13^, ^14^ Although its expression is predominantly extrahepatic, superimposed CA‐VB deficiency in a mouse model of CA‐VA deficiency exaggerated hyperammonemia suggesting that it has a role to play, albeit less important than CA‐VA, in handling ammonia flux.^13^ Another important clinical observation to refer to in this context is that despite the potent inhibition of CA‐VA caused by topiramate, a commonly prescribed anti‐epileptic medication, the complication of hyperammonemia is not frequently encountered clinically though it has been reported in the literature.^15^, ^16^, ^17^, ^18^ We argue that the role of CA‐VB as a potential genetic modifier in patients with CA‐VA deficiency deserves further study. Bicarbonate generated through nonenzymatic reactions in the mitochondria, even in the state of deficient mitochondrial carbonic anhydrases, may also contribute to mitigating hyperammonemia in these cases.^14^

Besides the above discussed cases of recurrent hyperammonemia, the clinical outcomes experienced in our cohort have not always been as favorable as previous reports have suggested.^2^, ^4^, ^7^, ^8^ Developmental delay was seen in 7/18 (39%) patients which was severe in 2 of them, 3 patients had microcephaly, and 7/18 (39%) patients suffered failure to thrive. Although the possibility cannot be excluded of a co‐inherited genetic condition(s) causing microcephaly in these consanguineous families where no extensive genetic evaluation was carried out, the developmental delay encountered in some patients cannot be explained consistently by the degree or duration of hyperammonemia. In fact, the patient with the shortest duration of hyperammonemia, patient 18, had global developmental delay while the patient with the longest duration of hyperammonemia, patient 6, continues to display a normal developmental outcome (Table 1). It is of clinical relevance to note that during the hyperammonemic episode, all patients with available lactate level had lactic acidemia, the majority had HyperCKemia, a few patients had documented hypoglycemia while one patient had a metabolic stroke. These complications may have resulted from energy deficiency that may have been secondary to the impact of deficient bicarbonate supply on the function of pyruvate carboxylase and/or 3‐methylcrotonyl‐CoA carboxylase. It is not clear why some patients have been more vulnerable to these complications than others in this cohort. Further reports and longitudinal studies are needed before the association of these complications with this diagnosis can be reliably validated.

In conclusion, this study reports the largest cohort of patients with CA‐VA deficiency, and it emphasizes the need to practice caution with long‐term prognostic implications of this diagnosis. The remarkable intrafamilial and interfamilial variability displayed in this cohort highlights the limitations of the current understanding of the pathomechanisms involved in this disorder, and calls for further evaluation of the possible role of genetic modifiers in this condition.

## AUTHOR CONTRIBUTIONS

KT contributed to the original draft, concept and design, manuscript development, and has approved the submitted version. NH, AB, and FJ contributed to the analysis and interpretation of data, critical revision of the manuscript, and patient management. AH contributed to the data gathering and manuscript critical revision. EA provided the neuroradiology features and images and critical revision of the manuscript. FM contributed to the critical revision of the manuscript, manuscript development, patient management, and has approved the submitted version.

## CONFLICT OF INTEREST STATEMENT

The authors declare no conflicts of interest.

## ETHICS STATEMENT

This study was conducted in agreement with the Declaration of Helsinki and the standard ethical principles governing patients care.

## INFORMED CONSENT

No patient identifying information is included in this article. All investigations and interventions described the patients were subjected to were part of standard clinical care, and patients were consented appropriately for genetic testing.

## ANIMAL RIGHTS

This article does not contain any studies involving human or animal subjects.

## Data Availability

All details pertaining to the data presented in this manuscript are available for anonymous review.
